# Real-World Effectiveness of Adjuvant Oxaliplatin Chemotherapy in Stage III Colon Cancer: A Controlled Interrupted Time Series Analysis

**DOI:** 10.3389/fphar.2021.693009

**Published:** 2021-06-29

**Authors:** Wen-Kuan Huang, Hung-Chih Hsu, Shu-Hao Chang, Wen-Chi Chou, Pei-Hung Chang, Sum-Fu Chiang, John Wen-Cheng Chang, Jen-Shi Chen, Tsai-Sheng Yang, Lai-Chu See

**Affiliations:** ^1^ Division of Hematology-Oncology, Department of Internal Medicine, Chang Gung Memorial Hospital at Linkou, Taoyuan, Taiwan; ^2^ College of Medicine, Chang Gung University, Taoyuan, Taiwan; ^3^ Department of Oncology-Pathology, Karolinska Institutet, Stockholm, Sweden; ^4^ Department of Public Health, College of Medicine, Chang Gung University, Taoyuan, Taiwan; ^5^ Division of Hematology-Oncology, Department of Internal Medicine, Chang Gung Memorial Hospital at Keelung, Keelung, Taiwan; ^6^ Division of Colon and Rectal Surgery, Chang Gung Memorial Hospital at Linkou, Taoyuan, Taiwan; ^7^ Division of Rheumatology, Allergy and Immunology, Department of Internal Medicine, Chang Gung Memorial Hospital at Linkou, Taoyuan, Taiwan; ^8^ Biostatistics Core Laboratory, Molecular Medicine Research Center, Chang Gung University, Taoyuan, Taiwan

**Keywords:** oxaliplatin, adjuvant chemotherapy, stage III colorectal cancer, interrupted time series study, mortality

## Abstract

**Background:** The real-world effectiveness of oxaliplatin in stage III colon cancer has not been determined in a large-scale population. We aimed to assess the real-world impact of adjuvant oxaliplatin treatment on the survival of these patients.

**Methods:** Based on Taiwan cancer registry, we evaluated 17,801 patients with resected stage III colon cancer, including 14,168 patients receiving adjuvant chemotherapy and 3,633 not receiving adjuvant chemotherapy as the control group between 2004 and 2014. We used the controlled interrupted time-series analysis to assess the three-year disease-free survival and five-year overall survival rates before (2004–2008) and after (2009–2014) the addition of oxaliplatin.

**Results:** The introduction of oxaliplatin was associated with no significant improvement in the slopes (per half-year) of the three-year disease-free survival rate (0.2%, 95% CI: −1.7∼2.2%) and five-year overall survival rate (0.6%, 95% CI: −1.8∼3%). The patients receiving oxaliplatin-based chemotherapy also showed no significant increase in the slopes (per half-year) of the three-year disease-free survival rate (0.6%, 95% CI: −1.4∼2.6%) and five-year overall survival rate (1%, 95% CI: −1.5∼3.5%). The nonsignificant results were consistent across subgroup analyses of age (<70 vs. ≥70 years), recurrence risk (T1-3 or N1 vs. T4 or N2), and cycle of oxaliplatin use (≤6 vs. >6). However, oxaliplatin-based chemotherapy significantly increased the slope (per half-year) of the five-year OS (2%, 95% CI: 0.2∼3.8%) for patients in the high-risk group (T4 or N2). The present results were robust in several sensitivity analyses.

**Conclusion:** Among real-world patients with stage III colon cancer, the introduction of oxaliplatin does not yield a significant improvement in survival. Future work should identify the subpopulation(s) of patients who benefit significantly from the addition of oxaliplatin.

## Introduction

Colon cancer is the fourth most common type of cancer and one of the leading causes of cancer related deaths worldwide, with more than 1,000,000 new cases and approximately 550,000 deaths recorded in 2018 (Bray et al., 2018). For stage III colon cancer, fluoropyrimidine-based chemotherapy is the standard treatment following surgical resection. Compared to surgery alone, fluoropyrimidine-based chemotherapy provides an absolute survival benefit of 10.3% at eight years (Sargent et al., 2009). In three randomized clinical trials, the addition of oxaliplatin to a fluorouracil and leucovorin regimen or oral capecitabine regimen has consistently shown a 20% reduction in disease recurrence (Andre et al., 2009; Haller et al., 2011; Yothers et al., 2011). The MOSAIC and XELOXA studies reported a significant absolute survival benefit of 8.1% at 10 years [67.1 vs. 59%, hazard ratio (HR): 0.80, 95% CI: 0.66∼0.96, *p* = 0.016] and 6% at 7 years (67 vs. 73%, HR: 0.83, 95% CI 0.70∼0.99, *p* = 0.036), respectively (Andre et al., 2015; Schmoll et al., 2015). A pooled analysis of 12,223 patients in five randomized clinical trials further confirmed the positive impact of oxaliplatin on survival (Shah et al., 2016).

Observational studies assessing the effectiveness of oxaliplatin-based adjuvant chemotherapy have reported conflicting findings (Sanoff et al., 2012a; Healey et al., 2013; Kim et al., 2014; Brungs et al., 2018). Some studies reported no significant survival benefit from the addition of oxaliplatin (Healey et al., 2013; Kim et al., 2014). Meanwhile, a study analyzing five community-based cohorts found a significant benefit in three-year OS for only two cohorts (Sanoff et al., 2012a). These conflicting findings could be due to smaller sample sizes; fewer events or deaths; shorter follow-up periods; or patient selection, if the patients receiving oxaliplatin tended to be younger, less frail, and have fewer comorbidities (Van Gils et al., 2012; Kim et al., 2016). Moreover, recent evidence indicates the non-inferiority of shorter duration (3 months) adjuvant oxaliplatin for low-risk stage III colon cancer patients (Grothey et al., 2018; Lieu et al., 2019). Therefore, there remains an unmet need to assess the real-world effectiveness of oxaliplatin based on age, recurrence risk, and treatment duration.

Age, severity of illness, and adherence to adjuvant treatment frequently differ between randomized trials and clinical practice settings (Batra et al., 2020). Real-world studies using high-quality, well-maintained national registries have emerged to provide a reality check for efficacy results from clinical trials (Booth et al., 2019). Although observational studies are subject to nonrandomization and unmeasured confounding, a robust approach such as quasi-experimental design allows for rigorous evaluation of intervention effectiveness (Venkataramani et al., 2016; Handley et al., 2018). The controlled interrupted time series (CITS) is one of the strongest quasi-experimental designs and can provide high-quality evidence (Schmidt, 2017; Lopez Bernal et al., 2019), making it valuable for medical research (Kontopantelis et al., 2015).

In February 2009, the Taiwanese national health insurance system started to reimburse oxaliplatin as part of adjuvant chemotherapy for patients with stage III colon cancer. This study aimed to assess the real-world impact of adjuvant oxaliplatin on survival in patients with resected stage III colon cancer. Toward this goal, we compared the three-year disease-free survival (DFS) rate and five-year overall survival (OS) rate before (2004–2008) and after (2009–2014) the interruption time of oxaliplatin becoming reimbursable in Taiwan, among patients with resected stage III colon cancer receiving adjuvant chemotherapy. The control group included those who did not receive adjuvant chemotherapy.

## Methods

### Study Design

In this CITS study, we compared trends in the three-year DFS and five-year OS following the start of oxaliplatin reimbursement (the “intervention”) to pre-intervention trends in these metrics (the counterfactual). Patients who did not receive adjuvant chemotherapy were used as the control group. Inclusion of the control series allowed for the adjustment for other time-varying confounders or cointerventions, which resulted in an unbiased estimate of the effect of the intervention (Lopez Bernal et al., 2018). The present study was approved by the Institutional Review Board of the Chang Gung Medical Foundation (201802145B0). The need for informed consent was waived due to the retrospective nature of the present study.

### Study Population

The subjects were patients newly diagnosed with stage III colon cancer between January 1, 2004, and December 31, 2014. The inclusion criterion was surgical resection within six months of diagnosis. Patients diagnosed with non-adenocarcinoma histology and those missing data on sex and birth year were excluded. To reduce the variation in compliance with standard care, we also excluded patients who received adjuvant chemotherapy more than 6 months after surgical resection (Supplementary Material S1).

### Data Sources

The Taiwan Cancer Registry is a national database that has been prospectively collecting data on patients with newly diagnosed cancer since 1979. The completeness of patient registration is 94.3–98.3% from 2004 to 2017 (National Health Insurance, 2017–2018; Taiwan Cancer Registry/Database, 2020). The National Health Insurance Research Database (NHIRD) was created for research purposes and derives data from the original claims data of the NHI program, which covers 99.7% Taiwan’s 23 million people. Dates and causes of death were obtained from the National Death Registry (Lu et al., 2000). Using data links between the Taiwan Cancer Registry, NHIRD, and Death Registry, we were able to longitudinally assess each patient from initial cancer diagnosis to survivorship.

### Outcomes

The outcomes of interest were the three-year DFS rate and the five-year OS rate, which are commonly used to evaluate outcomes when studying the effect of adjuvant treatment on colon cancer (Sargent et al., 2007). The follow-up time for the outcomes started on the date of surgical resection. We calculated the three-year DFS and five-year OS rates for each half-year cohort using the Kaplan-Meier method. The end of follow-up in this study was December 31, 2017; therefore, the cut-off for patient enrollment was December 31, 2014 for the three-year DFS rate and December 31, 2012 for the five-year OS rate. Hence, there were 10 three-year DFS rates and 10 five-year OS rates for each half-year cohort before the interruption time (year 2009), and there were 12 three-year DFS rates and eight five-year OS rates for each half-year cohort after the interruption time.

### Covariates

Patient-level covariates including age, sex, enrollee category, income, tumor location, tumor grade, pathological tumor and node staging, surgery type, surgical margins, comorbidities, and medications related to mortality [e.g., aspirin (Liao et al., 2012), metformin (Coyle et al., 2016), and statin (Voorneveld et al., 2017)] were obtained from claims data during the 12-month period prior to the diagnosis of colon cancer.

### Statistical Analysis

Categorical variables were expressed as frequencies and percentages, while continuous variables were expressed as the mean, SD, median and interquartile range (IQR). We used a segmented regression model of half-yearly data to estimate the level change and slope change of the three-year DFS rates and five-year OS rates following the introduction of oxaliplatin, for the adjuvant treatment and control groups. Due to the gradually changing survival of patients with colon cancer (Arnold et al., 2017), we assumed the trends in each segment to be linear. The time unit in this analysis was half-years. The regression model for this CITS analysis was adopted from the equation by Bernal et al. (2017) (Supplementary Material S2). We assessed whether there were differences in the outcome trends before and after the introduction of oxaliplatin and whether these differences varied for the adjuvant treatment and control groups, i.e., the difference-in-differences of slopes. The regression coefficients of the level change or slope change are provided with their 95% CI. For the post-oxaliplatin period, we assessed not only the overall effect of the whole adjuvant treatment group, but also of the oxaliplatin and the fluoropyrimidine alone groups separately. Autocorrelation was assessed using the Durbin-Watson statistic. To improve the comparability between the adjuvant treatment group and the control group, we used a generalized boosted regression model to obtain the stabilized inverse probability of treatment weighting of propensity score (SIPTW) for each half-yearly cohort (Supplementary Material S2) (Xu et al., 2010), Standardized mean differences (SMD) were determined to measure the covariate balance for each half-yearly cohort, in which an absolute of SMD >0.1 was indicative of meaningful imbalance. We also conducted CITS analyses on the predefined subgroup analyses: age (<70 vs. ≥70 years), risk of recurrence (T1-3N1 vs. T4 or N2), and number of oxaliplatin cycles (≤6 vs. >6). All statistical analyses were performed using SAS statistical software version 9.4 (SAS Institute Inc., Cary, NC, United States). A *p* value < 0.05 was considered statistically significant.

### Sensitivity Analysis

We conducted four sensitivity analyses to assess the robustness of the main results with various study designs. First, we excluded patients with a past history of cancers other than colon cancer because past cancer history might affect survival estimates. Second, we set a one-year transition period to exclude the period during which reimbursement rapidly increased. Third, some patients may have self-paid for oxaliplatin as part of adjuvant chemotherapy before it became reimbursable, and these patients would not be recognizable in the NHIRD; therefore, we excluded these patients by censoring those who received biweekly fluorouracil-based adjuvant chemotherapy in the pre-intervention period. Fourth, we conducted joint point regression to detect abrupt changes of longitudinal outcomes without indicating the time point (Kim et al., 2000).

## Results

### Patient Characteristics

After excluding 1,311 of the 19,112 patients identified, 17,801 patients were included in the analysis (Supplementary Material S1). Of these, 14,168 (79.6%) and 3,633 (20.4%) patients did and did not receive adjuvant chemotherapy after resection, respectively. The median age of the control group was 75 years, whereas that of the adjuvant treatment group was 65 years. In the control group, patients in the post-intervention period were older; had more laparoscopic surgery; had more dyslipidemia; and had more aspirin, metformin, and statin use than those in the pre-intervention period. Similarly, patients in the intervention period in the adjuvant chemotherapy group had more laparoscopic surgeries than those in the pre-intervention period, but the distribution of other clinicopathologic covariates was generally similar between the pre-intervention and post-intervention periods (Table 1). After SIPTW, the covariates were relatively balanced across the study groups (Supplementary Material S3). Supplementary Material S4 shows the number and distribution of patients receiving fluoropyrimidine alone and oxaliplatin-based chemotherapy each year. The number of eligible patients (stage III colon cancer) per half-year was around 500 in 2004 and doubled in 2014. Oxaliplatin use increased in 2008 and stayed at 50–60% among all patients with stage III colon cancer in years thereafter.

**TABLE 1:** Characteristics of demographic, tumor, comorbidity, medication use among patients with stage III colon cancer before (2004–2008) and after (2009–2014) introduction of oxaliplatin, before SIPTW

	No adjuvant				Adjuvant			
	Overall (*n* = 3633)	Pre-intervention (*n* = 1337)	Post-intervention (*n* = 2296)	SMD	Overall (*n* = 14168)	Pre-intervention (*n* = 4293)	Post-intervention (*n* = 9875)	SMD
Age				0.13				0.03
Median (IQR)	75 (19)	73 (20)	76 (20)		65 (19)	65 (19)	65 (18)	
Mean (SD)	71.75 (14.11)	70.22 (14.23)	72.64 (13.96)		64.04 (12.95)	63.63 (13.12)	64.22 (12.88)	
Range	15–102	17–99	15–102		13–99	15–95	13–99	
< 50	285 (7.84%)	118 (8.83%)	167 (7.27%)		1854 (13.09%)	615 (14.33%)	1239 (12.55%)	
50–59	460 (12.66%)	186 (13.91%)	274 (11.93%)		3240 (22.87%)	968 (22.55%)	2272 (23.01%)	
60–69	580 (15.96%)	227 (16.98%)	353 (15.37%)		3784 (26.71%)	1095 (25.51%)	2689 (27.23%)	
≥ 70	2308 (63.53%)	806 (60.28%)	1502 (65.42%)		5290 (37.34%)	1615 (37.62%)	3675 (37.22%)	
Sex				−0.02				0
Men	1936 (53.29%)	723 (54.08%)	1213 (52.83%)		7632 (53.87%)	2306 (53.72%)	5326 (53.93%)	
Women	1697 (46.71%)	614 (45.92%)	1083 (47.17%)		6536 (46.13%)	1987 (46.28%)	4549 (46.07%)	
Enrollee category				0.15				0.05
EC1	264 (7.27%)	128 (9.57%)	136 (5.92%)		1152 (8.13%)	388 (9.04%)	764 (7.74%)	
EC2	850 (23.4%)	308 (23.04%)	542 (23.61%)		4034 (28.47%)	1186 (27.63%)	2848 (28.84%)	
EC3	1452 (39.97%)	533 (39.87%)	919 (40.03%)		5550 (39.17%)	1717 (40%)	3833 (38.82%)	
EC4	1067 (29.37%)	368 (27.52%)	699 (30.44%)		3432 (24.22%)	1002 (23.34%)	2430 (24.61%)	
Income (NT$)				0.05				0.11
Dependent	1270 (34.96%)	448 (33.51%)	822 (35.8%)		4746 (33.5%)	1350 (31.45%)	3396 (34.39%)	
<15,000	838 (23.07%)	304 (22.74%)	534 (23.26%)		2642 (18.65%)	799 (18.61%)	1843 (18.66%)	
15,000–24,999	1143 (31.46%)	437 (32.69%)	706 (30.75%)		4262 (30.08%)	1443 (33.61%)	2819 (28.55%)	
≥ 25,000	382 (10.51%)	148 (11.07%)	234 (10.19%)		2518 (17.77%)	701 (16.33%)	1817 (18.4%)	
Tumor location				0.05				0.12
Left side	1665 (45.83%)	622 (46.52%)	1043 (45.43%)		6997 (49.39%)	2163 (50.38%)	4834 (48.95%)	
Right side	1520 (41.84%)	545 (40.76%)	975 (42.47%)		5270 (37.2%)	1632 (38.02%)	3638 (36.84%)	
Rectosigmoid	380 (10.46%)	136 (10.17%)	244 (10.63%)		1727 (12.19%)	441 (10.27%)	1286 (13.02%)	
Unspecified	68 (1.87%)	34 (2.54%)	34 (1.48%)		174 (1.23%)	57 (1.33%)	117 (1.18%)	
Tumor grade				0.04				0.09
Well or moderately differentiated	2877 (79.19%)	1062 (79.43%)	1815 (79.05%)		12226 (86.29%)	3718 (86.61%)	8508 (86.16%)	
Poorly differentiated	456 (12.55%)	179 (13.39%)	277 (12.06%)		1591 (11.23%)	438 (10.2%)	1153 (11.68%)	
Unknown	300 (8.26%)	96 (7.18%)	204 (8.89%)		351 (2.48%)	137 (3.19%)	214 (2.17%)	
pT stage (AJCC 6th and 7th)				0.26				0.13
1	49 (1.35%)	20 (1.5%)	29 (1.26%)		409 (2.89%)	85 (1.98%)	324 (3.28%)	
2	193 (5.31%)	77 (5.76%)	116 (5.05%)		886 (6.25%)	213 (4.96%)	673 (6.82%)	
3	2260 (62.21%)	899 (67.24%)	1361 (59.28%)		10008 (70.64%)	3193 (74.38%)	6815 (69.01%)	
4	773 (21.28%)	267 (19.97%)	506 (22.04%)		2789 (19.69%)	776 (18.08%)	2013 (20.38%)	
0 + Unknown	358 (9.85%)	74 (5.53%)	284 (12.37%)		76 (0.54%)	26 (0.61%)	50 (0.51%)	
pN stage (AJCC 6th and 7th)				0.26				0
1	2187 (60.2%)	832 (62.23%)	1355 (59.02%)		9185 (64.83%)	2789 (64.97%)	6396 (64.77%)	
2	1094 (30.11%)	436 (32.61%)	658 (28.66%)		4912 (34.67%)	1482 (34.52%)	3430 (34.73%)	
0 + Unknown	352 (9.69%)	69 (5.16%)	283 (12.32%)		71 (0.50%)	22 (0.51%)	49 (0.5%)	
Surgery type				0.53				0.6
Open	2953 (81.28%)	1185 (88.63%)	1768 (77%)		11491 (81.11%)	4022 (93.69%)	7469 (75.64%)	
Laprascopic	405 (11.15%)	22 (1.65%)	383 (16.68%)		2330 (16.45%)	133 (3.1%)	2197 (22.25%)	
Unknown	275 (7.57%)	130 (9.72%)	145 (6.32%)		347 (2.45%)	138 (3.21%)	209 (2.12%)	
Surgical margins				0.13				0.06
No	2978 (81.97%)	1140 (85.27%)	1838 (80.05%)		13544 (95.6%)	4119 (95.95%)	9425 (95.44%)	
Yes	120 (3.3%)	36 (2.69%)	84 (3.66%)		368 (2.6%)	78 (1.82%)	290 (2.94%)	
Unknown	535 (14.73%)	161 (12.04%)	374 (16.29%)		256 (1.81%)	96 (2.24%)	160 (1.62%)	
Charlson comorbidity score				0.13				0.08
0	599 (16.49%)	259 (19.37%)	340 (14.81%)		3492 (24.65%)	1142 (26.6%)	2350 (23.8%)	
1	679 (18.69%)	267 (19.97%)	412 (17.94%)		3453 (24.37%)	1074 (25.02%)	2379 (24.09%)	
≥ 2	2355 (64.82%)	811 (60.66%)	1544 (67.25%)		7223 (50.98%)	2077 (48.38%)	5146 (52.11%)	
Comorbidity								
Prior cancer history	519 (14.29%)	216 (16.16%)	303 (13.2%)	−0.08	1305 (9.21%)	485 (11.3%)	820 (8.3%)	−0.1
Ischemic heart disease	1756 (48.33%)	612 (45.77%)	1144 (49.83%)	0.08	5007 (35.34%)	1490 (34.71%)	3517 (35.62%)	0.02
Stroke	1014 (27.91%)	329 (24.61%)	685 (29.83%)	0.12	2158 (15.23%)	612 (14.26%)	1546 (15.66%)	0.04
Diabetes mellitus	1549 (42.64%)	538 (40.24%)	1011 (44.03%)	0.08	5110 (36.07%)	1438 (33.5%)	3672 (37.18%)	0.08
Hypertension	2607 (71.76%)	924 (69.11%)	1683 (73.3%)	0.09	8314 (58.68%)	2382 (55.49%)	5932 (60.07%)	0.09
Dyspilidemia	1736 (47.78%)	538 (40.24%)	1198 (52.18%)	0.24	6638 (46.85%)	1729 (40.27%)	4909 (49.71%)	0.19
Chronic kidney disease	1477 (40.66%)	482 (36.05%)	995 (43.34%)	0.15	4173 (29.45%)	1147 (26.72%)	3026 (30.64%)	0.09
Chronic liver disease	1508 (41.51%)	534 (39.94%)	974 (42.42%)	0.05	5638 (39.79%)	1580 (36.8%)	4058 (41.09%)	0.09
Medications								
Aspirin	1479 (40.71%)	482 (36.05%)	997 (43.42%)	0.15	4028 (28.43%)	1071 (24.95%)	2957 (29.94%)	0.11
Metformin	801 (22.05%)	239 (17.88%)	562 (24.48%)	0.16	2610 (18.42%)	627 (14.61%)	1983 (20.08%)	0.15
Statin	849 (23.37%)	198 (14.81%)	651 (28.35%)	0.33	3009 (21.24%)	617 (14.37%)	2392 (24.22%)	0.25

SIPTW, stabilized inverse probability of treatment weighting; SMD, standardized mean differences

### Trends in Three-Year DFS and Five-Year OS

#### Overall Cohort

**FIGURE 1:**
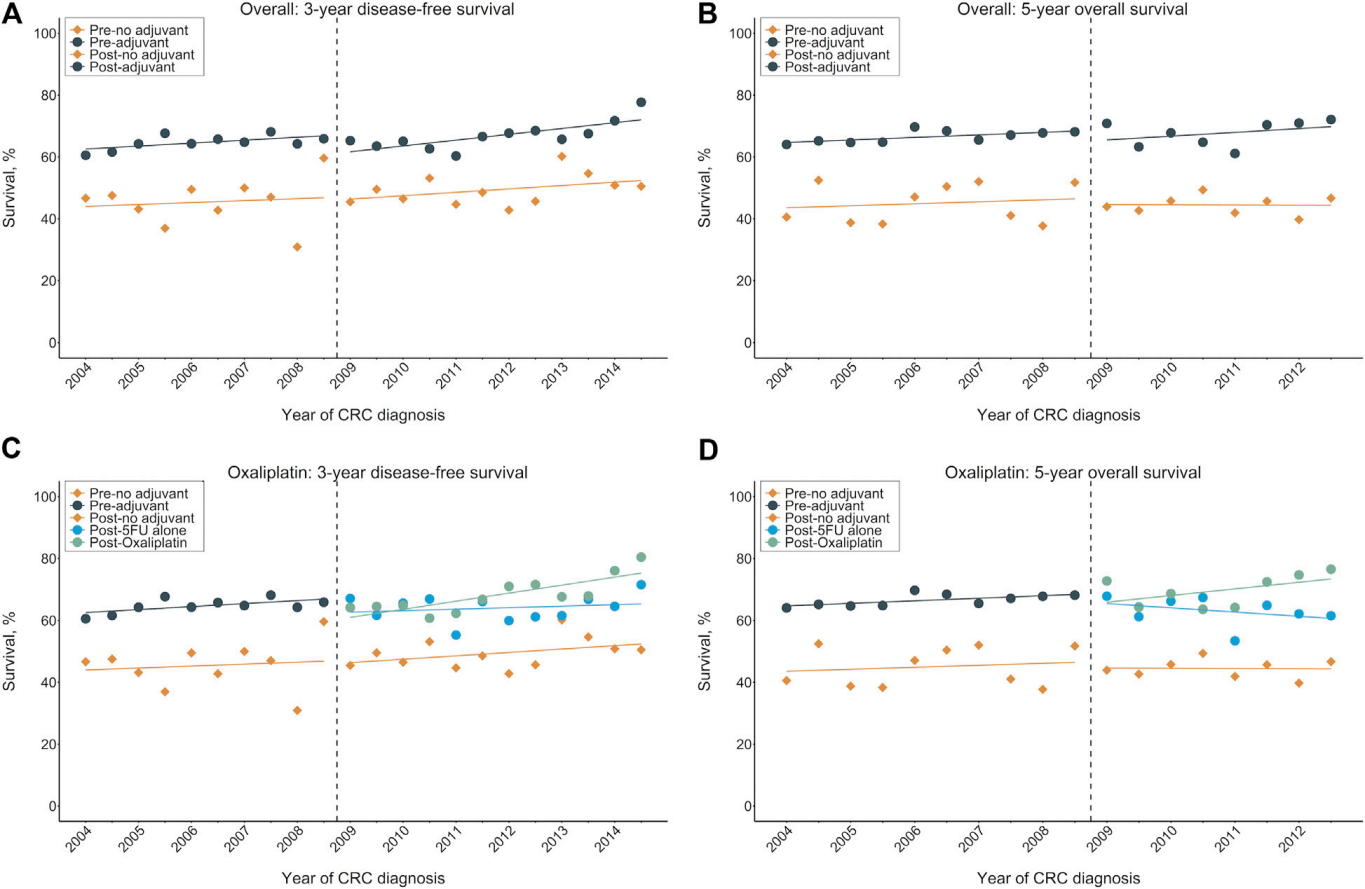
Survival outcomes by calendar year before and after oxaliplatin reimbursement in the entire population. **(A)** Three-year DFS rates for overall cohorts, **(B)** Five-year OS rates for overall cohorts, **(C)** Three-year DFS rates for oxaliplatin cohort, **(D)** Five-year OS rates for oxaliplatin cohort. The vertical broken lines delineate the intervention time [between quarter 4 (Q4) 2008 and Q1 2009]. DFS: disease-free survival; OS: overall survival.

**FIGURE 2:**
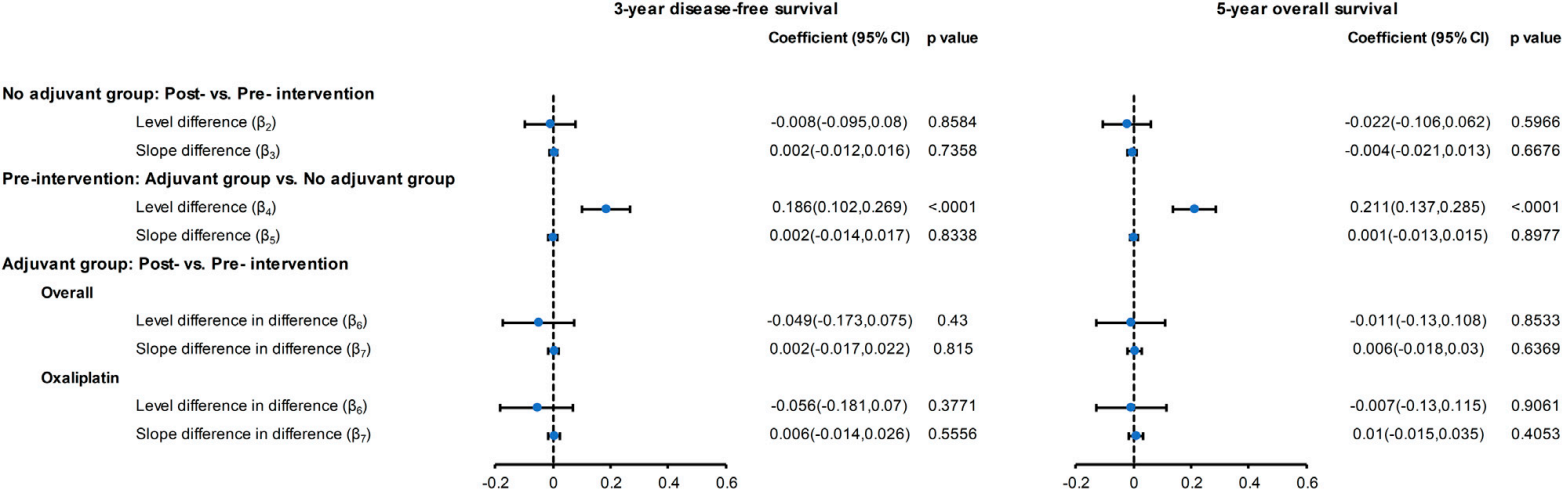
Forest plot of the regression coefficients for the CITS for the overall cohort and for the oxaliplatin cohort. Please refer to eMethod 1 in the Supplement for explanation of the regression coefficients (*β*_*2*_ *–β*_*7*_).

Figure 1 displays the three-year DFS and five-year OS before and after oxaliplatin became reimbursable for the entire population. The controlled time-series analyses (Figure 2) revealed that for the no adjuvant group, there was no significant change in the level (*β*_*2*_) or slope (*β*_*3*_) of the three-year DFS and five-year OS in the post-intervention period compared to the pre-intervention period. In the pre-intervention period, patients who received adjuvant chemotherapy showed a significant level increase (*β*_*4*_) in the three-year DFS (18.6%, 95% CI: 10.2∼26.9%, *p* < 0.001) and five-year OS (21.1%, 95% CI, 13.7∼28.5%, *p* < 0.001) compared to those who did not receive adjuvant chemotherapy. When considering the slope difference (*β*_*5*_) between those with and without adjuvant treatment in the pre-intervention period, no significant difference was observed for both the three-year DFS (0.2% per half-year, 95% CI: −1.4∼1.7%, *p* = 0.8338) and five-year OS (0.1% per half-year, 95% CI: −1.3∼1.5%, *p* = 0.897). In the post-intervention period, the introduction of oxaliplatin reimbursement was not associated with a significant level change (*β*_*6*_) in the three-year DFS (−4.9% per half-year, 95% CI: −17.3∼7.5%, *p* = 0.430) and five-year OS (−1.1% per half-year, 95% CI: −13∼10.8%, *p* = 0.853) in the adjuvant chemotherapy group compared to the control group. There was no significant slope change (*β*_*7*_) in the three-year DFS (0.2% per half-year, 95% CI: −1.7∼2.2%, *p* = 0.815) and five-year OS (0.6% per half-year, 95% CI: −1.8∼3.0%, *p* = 0.636) in the adjuvant chemotherapy group compared to the control group. The values of the Durbin-Watson statistic were 2.514 and 2.284 for the DFS and OS, respectively, indicating no significant autocorrelation.

Furthermore, when only patients who received oxaliplatin-based adjuvant chemotherapy in the post-intervention period were evaluated, there was no significant level change (*β*_*6*_) in the three-year DFS (−5.6% per half-year, 95% CI: −18.1∼7%, *p* = 0.377) or five-year OS (−0.7% per half-year, 95% CI: −13∼11.5%, *p* = 0.906). Similarly, the oxaliplatin-based chemotherapy group showed an nonsignificant slope change (*β*_*7*_) in the three-year DFS (0.6% per half-year, 95% CI: −1.4∼2.6%, *p* = 0.555) and five-year OS (1% per half-year, 95% CI, −1.5∼3.5%, *p* = 0.405) compared to the non-adjuvant treatment group (Figure 2).

#### Subgroup Analyses

**FIGURE 3:**
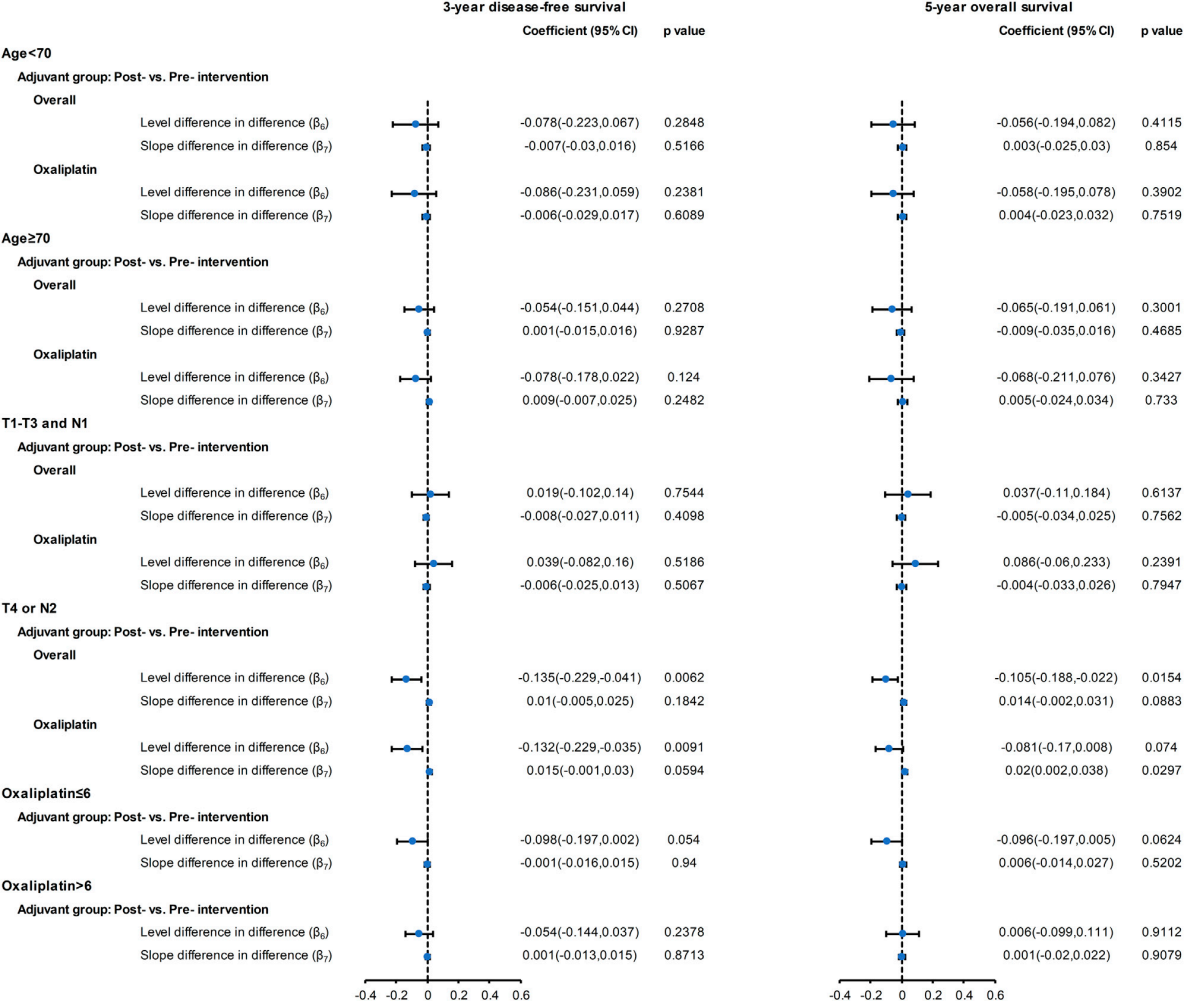
Forest plot of the regression coefficients for the CITS for the overall cohort and for the oxaliplatin cohort with subgroups: aged <70 years; aged ≥70 years; T1-3 and N1; T4 or N2; ≤ 6 oxaliplatin cycles; >6 oxaliplatin cycles. Please refer to the eMethod 1 in the Supplement for the explanation of the regression coefficients (*β*_*6*_ *,* β_*7*_).

The results of CITS in the subgroup analyses by age, cancer stage, and cycle of oxaliplatin use are shown in Figure 3 and Supplementary Material S5–S8. The results of subgroup analysis by age and by cycle of oxaliplatin use are similar to the results from the main analysis. However, among high-risk patients (T4 or N2), we observed a significant slope change (2%, 95% CI: 0.2∼3.8%, *p* = 0.029) in the five-year OS following introduction of oxaliplatin reimbursement.

### Sensitivity Analyses

**FIGURE 4:**
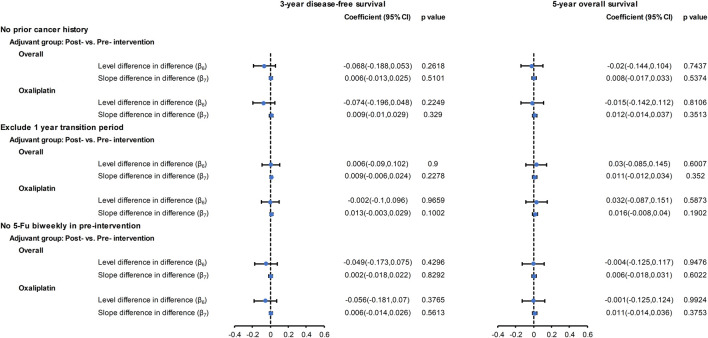
Forest plot of the regression coefficients for the CITS for the overall cohort and for the oxaliplatin cohort with sensitivity analyses: no prior cancer history, excluding one-year transition period, excluding biweekly fluoropyrimidine treatment before the intervention. Please refer to eMethod 1 in the Supplement for explanation of the regression coefficients (*β*_*6*_ *, β*_*7*_).

The results of the sensitivity analyses are provided in Figure 4, and Supplementary Materials S9, S10. The findings showed that the introduction of oxaliplatin had no significant effect on the three-year DFS and five-year OS after excluding patients with previous cancers (Supplementary Materials S9A,B), excluding data during the one-year transition period (Supplementary Materials S9C,D), and excluding patients who received biweekly fluorouracil-based chemotherapy (Supplementary Materials S9E,F). These findings supported the results of the main analysis. In joint point analysis, an abrupt change was detected for neither three-year DFS nor five-year OS for the no adjuvant, adjuvant, and oxaliplatin groups (Supplementary Material S11).

## Discussion

The potential survival benefit of the addition of oxaliplatin to adjuvant chemotherapy for colon cancer has only been evaluated in clinical trials. In this real-world large population analysis of patients with stage III colon cancer, the addition of oxaliplatin in the adjuvant setting did not significantly improve the three-year DFS and five-year OS rates. Patients who received oxaliplatin-based chemotherapy showed a slightly positive but nonsignificant secular change compared to the control group. Notably, we found that high-risk patients (T4 or N2) who received oxaliplatin-based chemotherapy had a significant 2% benefit in the trend change of their five-year OS. The addition of oxaliplatin had no significant impact on survival in the subgroup analyses by age, cancer stage, and number of oxaliplatin cycles. Consistent findings from several sensitivity analyses indicate the robustness of the main results.

Our finding that there is no significant survival benefit of adjuvant oxaliplatin use in colon cancer patients stands in contrast to results from previous landmark clinical trials. There are several possible explanations for the lack of real-world effectiveness. First, patients enrolled in the clinical trials were younger and had fewer comorbidities than our real-world cohort. In the clinical trials, the median age at diagnosis was 59–61 years (Andre et al., 2009; Haller et al., 2011; Yothers et al., 2011), whereas it was 75 years in the present study. Elderly patients with significant comorbidities were underrepresented in the clinical trials, which might account for discrepancies between the clinical trial results and our real-world findings. Second, the CITS model compared the effectiveness of adjuvant treatments between the pre- and post-intervention periods and also considered the population receiving no adjuvant chemotherapy, which was not accounted for in clinical trials. The three-year DFS for patients not receiving adjuvant chemotherapy has slightly improved over time, which partly explains the nonsignificant benefit of oxaliplatin-based chemotherapy. Third, in real-world practice, the dose of oxaliplatin and adherence to medication schedule were likely suboptimal, which may have led to underperformance in our study. For pharmacological interventions, an attenuated effect in real-world studies compared to clinical trials is not uncommon (Anglemyer et al., 2014). Given that the absolute survival benefit of oxaliplatin in clinical trials was only 6–8% in the long-term follow-up (Andre et al., 2015; Schmoll et al., 2015), we predict that the absolute benefit is more limited in real-world settings.

Notably, our results showed that for elderly patients (age ≥70 years), adjuvant chemotherapy had significant DFS and OS benefits compared to no adjuvant chemotherapy, which is consistent with previous findings (Sanoff et al., 2012b; Hoeben et al., 2013). However, the impact of adding oxaliplatin to adjuvant chemotherapy in elderly patients remains controversial (Mccleary et al., 2013; Haller et al., 2015). We also did not find a superior survival benefit of oxaliplatin for patients aged <70 years. Trends in the three-year DFS increased in both patients receiving oxaliplatin and those who did not receive adjuvant chemotherapy. There was a minimal difference in the slope in these trends between the two groups, resulting in the nonsignificant results.

In our study, only high-risk patients (T4 or N2) receiving oxaliplatin-based chemotherapy had a weak but significant increase of 2% per year in their five-year OS. A recent trial investigating the duration of adjuvant oxaliplatin use suggested a risk-based approach when determining adjuvant treatment (Grothey et al., 2018). High-risk patients tend to gain a more significant survival benefit than low-risk patients from six-month use of oxaliplatin. Similarly, we found a significant benefit in high-risk patients but not in low-risk patients.

The present study was not without limitations. First, although the populations in the pre- and post-intervention period were generally homogeneous, patients who received oxaliplatin-based chemotherapy or fluorouracil alone had different baseline characteristics than the pre-intervention population, which introduced selection bias. Although we used SIPTW to increase the homogeneity of patient-level data, the bias may not have been eliminated completely due to unmeasured confounders. Second, because the study period was more than 10 years, sequential use of active medications such as irinotecan, bevacizumab, and cetuximab may have acted as time-varying confounders affecting survival. That said, we used control groups in the time-series model, which allowed us to account for time-varying confounding. Third, some patients may have been treated with oxaliplatin and paid for it out of pocket before it became reimbursable. To address this, we conducted a sensitivity analysis to exclude patients who received biweekly fluorouracil before the intervention, which we used as a surrogate for oxaliplatin use. However, capecitabine may also be used in combination with oxaliplatin. Because data from the XELOXA study were first released in 2009, we reasoned that the number of patients treated with the combination of capecitabine and oxaliplatin before the intervention was limited. Fourth, we did not have data on adverse events and safety, which are also important concerns for oxaliplatin use. Fifth, our modeling approach may be sensitive to other unmeasured confounders, although the consistent results from several sensitivity analyses should minimize this possibility. Finally, this study was conducted in Taiwan, and therefore, the generalizability of these findings to other populations and settings needs to be confirmed.

## Conclusion

In this study with real-world setting, the introduction of oxaliplatin is not associated with a significant benefit in the three-year DFS and five-year OS of patients with stage III colon cancer. Consistent findings were seen regardless of age and the number of oxaliplatin cycles. Although we found a small but statistically significant improvement in five-year OS in high-risk patients (T4 or N2) receiving oxaliplatin-based chemotherapy, this finding needs to be confirmed in further studies. Our study indicated the importance of identifying patients with stage III colon cancer who could substantially benefit from oxaliplatin to improve prognosis.

## Data Availability

The datasets presented in this article are not readily available because the use of dataset needs the permission of the Ministry of Health and Welfare, Taiwan. Requests to access these datasets should be directed to Ministry of Health and Welfare, Taiwan.
