# Supplementary material for: Real-World Effectiveness of Adjuvant Oxaliplatin Chemotherapy in Stage III Colon Cancer: A Controlled Interrupted Time Series Analysis
Source: Front Pharmacol. 2021 Jun 29;12:693009. doi: 10.3389/fphar.2021.693009 (PMC8276019; doi:10.3389/fphar.2021.693009)
Supplement: Supplementary file 1 [file DataSheet1.docx]

Real-world effectiveness of adjuvant oxaliplatin chemotherapy in stage III colon cancer: a controlled interrupted time series analysis

**SUPPLEMENTAL MATERIAL**

**Supplement file 1.** Patient selection flowchart.

**Supplement file 2.** Supplemental methods

**Supplement file 3.** Characteristics of demographic, tumor, comorbidity, medication use among patients with stage III colon cancer before (2004-2008) and after (2009-2014) introduction of oxaliplatin, after SIPTW

**Supplement file 4.** (A) Number of patients with stage III colon cancer receiving different types of adjuvant treatment by calendar year. (B) The percentage distribution of patients with stage III colon cancer receiving different types of adjuvant treatment by calendar year.

**Supplement file 5.** Survival outcomes by calendar year before and after oxaliplatin reimbursement for subgroups. (A) Three-year DFS rates for age < 70 years old, (B) Three-year DFS rates for age > 70 years old, (C) Five-year OS rates for age < 70 years old, (D) Five-year OS rates for age > 70 years old, (E) Three-year DFS rates for T1-T3 and N1, (F) Three-year DFS rates for T4 or N2, (G) Five-year OS rates for T1-T3 and N1, (H) Five-year OS rates for T4 or N2, (I) Three-year DFS rates for oxaliplatin < 6 cycles, (J) Three-year DFS rates for oxaliplatin > 6 cycles, (K) Five-year OS rates for oxaliplatin < 6 cycles, (L) Five-year OS rates for oxaliplatin > 6 cycles.

**Supplement file 6.** The CITS result: Subgroup analysis by age

**Supplement file 7.** The CITS result: Subgroup analysis by stage

**Supplement file 8.** The CITS result: Subgroup analysis by cycle of oxaliplatin use

**Supplement file 9.** Survival outcomes by calendar year before and after oxaliplatin reimbursement, restricting to the following (sensitivity analysis). (A) Three-year DFS rates for those with no prior cancer history, (B) Five-year OS rates for those with no prior cancer history, (C) Three-year DFS rates excluding the one-year transition period, (D) Five-year OS rates excluding the one-year transition period, (E) Three-year DFS rates excluding patients receiving biweekly fluoropyrimidine treatment before the intervention, (F) Five-year OS rates excluding patients receiving biweekly fluoropyrimidine treatment before the intervention.

**Supplement file 10.** The CITS result: Sensitivity analysis

**Supplement file 11.** Joinpoint analysis of 3-year disease-free survival (DFS) and 5-year overall survival (OS) for patients with stage III colon cancer

**Supplement file 1. Patient selection flowchart.**

**
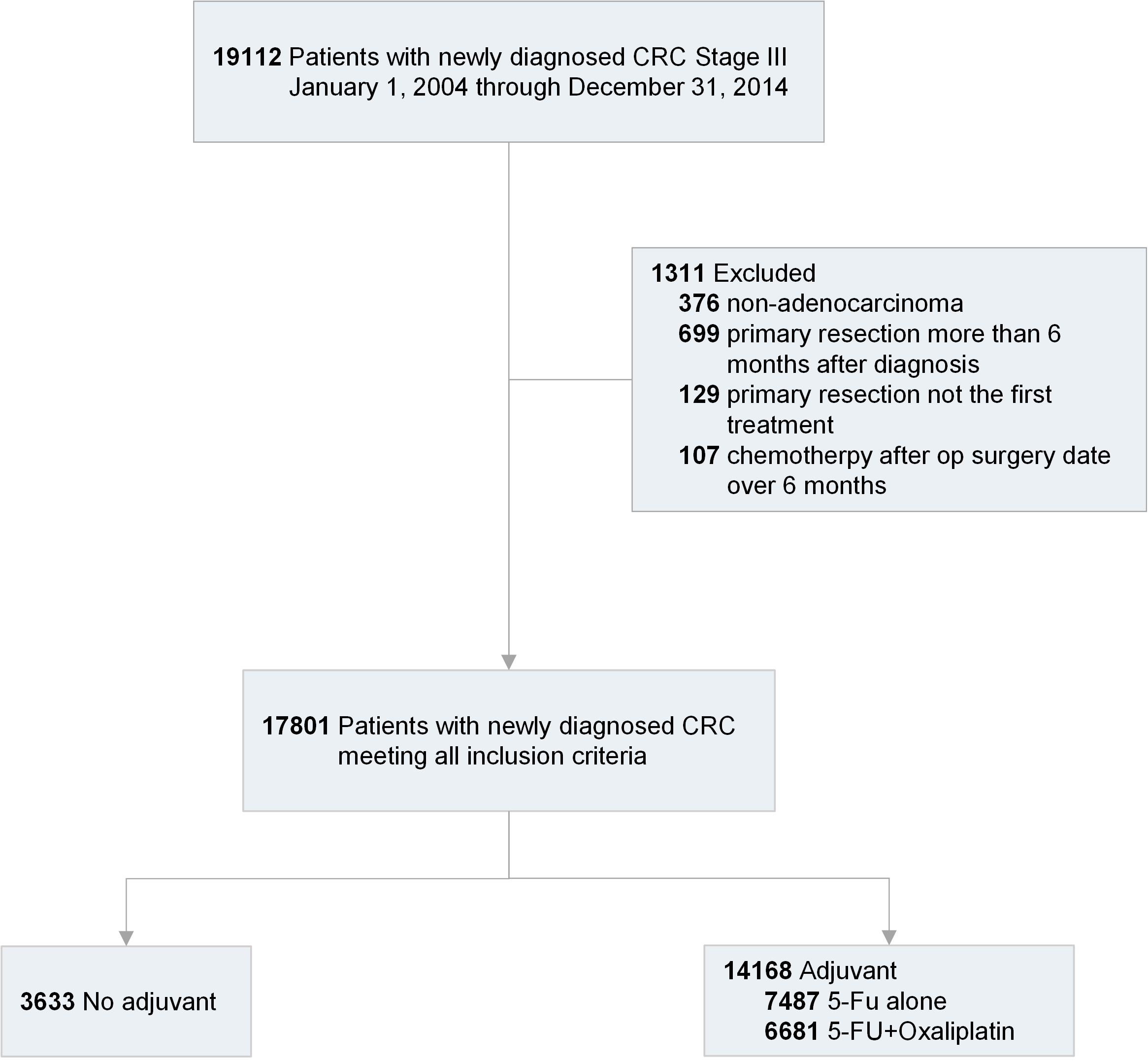
**

**Supplement file 2. Supplemental methods**

1. **Interrupted time-series (ITS) analysis**

An interrupted time-series (ITS) analysis includes one time-series (single ITS analysis) or multiple time-series (controlled interrupted time-series analysis, CITS) of observations on the same outcome (usually on an aggregate level), which are interrupted by an intervention at a known point in time. Hence, the intervention effect can be easily estimated by comparing the level change and slope change in the pre- and post-intervention periods using segmented regression analysis. The strengths of ITS include: (1) uses observational data in an aggregate format, (2) provides precise and easy-to-interpret graphical results, (3) uses readily available statistical methods (4) solves the problem of selection bias if every individual is accounted for when calculating the aggregated outcome variable^1^.

When there are two time-series of data (eFigure A)^2^, the segmented regression can be written as

$$Y_{T}=\text{ }\beta_{0}+\beta_{1}T+\beta_{2}X+\beta_{3}X\text{(}T-k)+\beta_{4}Z+\beta_{5}ZT+\beta_{6}ZX+\beta_{7}ZX(T-k\text{)}+\varepsilon_{T}$$

Let *Y_T_* = the aggregated outcome at each equally-spaced time point, t (three-year DFS or five-year OS for each half-year cohort in the present study), *T* = 0, 1,…t^th^ time point from the start of the series, *X* = 0 for pre-intervention and 1 for post-intervention, k = the last time point before the intervention (*k* =10 in the present study because oxaliplatin was introduced after the 10^th^ time point), Z = 0 for the control group (i.e., no adjuvant treatment group) and 1 for the treated group (i.e., adjuvant treatment group), *ε_T_* = the error.

Hence,

*β_0_* = the level of the outcome at the beginning of the pre-intervention period for the control group.

*β_1_* = the slope (change per equally spaced time duration) of the outcome in the pre-intervention period for the control group.

*β_2_* = the level change of the outcome immediately after the intervention started for the control group.

*β_3_* = the slope change of the outcome between the pre- and post-intervention periods for the control group.

*β_4_* = the level difference of the outcome at the beginning of the pre-intervention period between the control and treated groups.

*β_5_* = the slope difference of the outcome in the pre-intervention period between the control and treated groups.

*β_6_* = the difference between the treated and control groups in the level of the outcome immediately following the intervention.

*β_7_* = the difference between the treatment and control groups in the slope (trend) of the outcome after the intervention compared with pre-intervention (difference-in-difference of the slope).

| 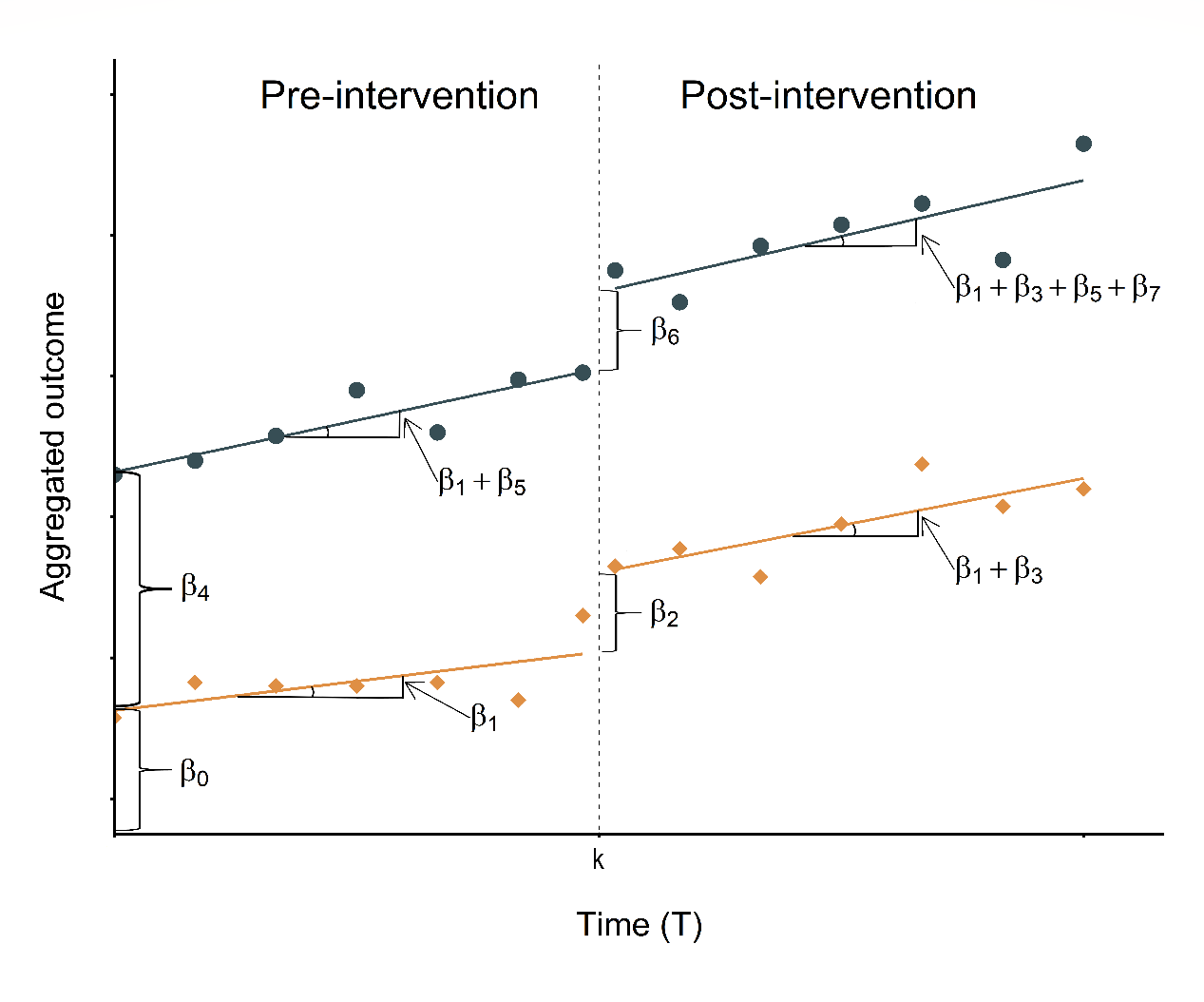 |
| --- |
| Figure A. Scatter plot for two time-series of aggregate outcome (controlled interrupted time series). The orange solid lines are the best fitted lines of *Yt* vs. *Tt* pre- or post-intervention for the control group. The grey solid lines are the best fitted lines of *Yt* vs. *Tt* pre- or post-intervention for the treated group. The vertical line indicates the time of the interruption. |

1. **Stabilized inverse probability of treatment weighting (SIPTW) of the propensity score using a generalized boosted regression model**

Rosenbaum et al^3^. first proposed the propensity score (PS) method in 1983 to mimic a clinical trial by balancing covariates between the treatment and control groups and then examining the effect of the treatment on various disease outcomes. PS weighting is the most common method to estimate the average treatment effects for a treated group (ATT) and the population (ATE)^4^. In ATT, a pseudo-control group (i.e., untreated group) with a distribution of covariates similar to that of the treated group is created by giving a weight of the estimated propensity score (*π_i_*) and dividing by one minus the propensity score (i.e., weight = *π_i_/(*1*-π_i_)*); the treated group is not weighted (i.e., weight = 1); *i* is for an individual patient. In ATE, the inverse probability of treatment weighting (IPTW) is often applied, and pseudo-treated groups and pseudo-control groups with covariate distributions similar to those of the total population are created. The treated group is weighted by the inverse of the estimated PS (π_i_) (i.e., weight = 1/*π_i_*), and the control group is weighted by the inverse of one minus the estimated PS (i.e., weight = 1/(1- *π*_i_)^5^. Unfortunately, IPTW can suffer from a high type I error because the sample sizes for both the pseudo-treated and pseudo-control groups are enlarged^4^. Xu et al proposed a solution to this problem by giving a stabilized IPTW (SIPTW), in which the proportion of the treated group and control group time the corresponding IPTW^6^. Hence, the sample size of the original data is maintained, and the variance of the main effect is appropriately estimated. In this study, we used the generalized boosted method (GBM) to obtain the SIPTW because GBM can automatically determine the best functions of covariates, including interactions or polynomial terms, to achieve optimal balance among study groups^6^. Another advantage of SIPTW obtained by GBM is that it is less affected by large weights.

**Supplementary References**

1. Bernal JL, Cummins S, Gasparrini A. Interrupted time series regression for the evaluation of public health interventions: A tutorial. Int. J. Epidemiol. 2017, 46(1): 348–355.

2. Turner SL, Karahalios A, Forbes AB, et al. Creating effective interrupted time series

graphs: Review and recommendations. *Research Synthesis Methods.* 2020,

12(1):106-117..

3. Rosenbaum PR, Rubin DB. The central role of the propensity score in observational studies for causal effects. *Biometrika.* 1983;70(1):41-55.

4. Brookhart MA, Wyss R, Layton JB, Sturmer T. Propensity score methods for confounding control in nonexperimental research. *Circ Cardiovasc Qual Outcomes.* 2013;6(5):604-611.

5. Austin PC. An Introduction to Propensity Score Methods for Reducing the Effects of Confounding in Observational Studies. *Multivariate Behav Res.* 2011;46(3):399-424.

6. Xu S, Ross C, Raebel MA, Shetterly S, Blanchette C, Smith D. Use of stabilized inverse propensity scores as weights to directly estimate relative risk and its confidence intervals. *Value Health.* 2010;13(2):273-277.

7. McCaffrey DF, Griffin BA, Almirall D, Slaughter ME, Ramchand R, Burgette LF. A tutorial on propensity score estimation for multiple treatments using generalized boosted models. Stat Med. 2013;32(19):3388–414.

**Supplementary file 3. Characteristics of demographic, tumor, comorbidity, medication use among patients with stage III colon cancer before (2004-2008) and after (2009-2014) introduction of oxaliplatin, after SIPTW**

|  |  | **No adjuvant** | |  |  | **Adjuvant** | |  |
| --- | --- | --- | --- | --- | --- | --- | --- | --- |
|  | **Overall (n=3383.16)** | **Pre-intervention (n=1201.52)** | **Post-intervention (n=2181.64)** | **SMD** | **Overall (n=13604)** | **Pre-intervention (n=4036.91)** | **Post-intervention (n=9567.12)** | **SMD** |
| **Age** |  |  |  | 0.07 |  |  |  | 0.00 |
| Median (IQR) | 67(23) | 67(21) | 67(24) |  | 67(19) | 67(19) | 67(19) |  |
| Mean (SD) | 66.67(14.07) | 66.1(13.54) | 66.98(14.36) |  | 64.95(12.41) | 64.99(12.39) | 64.94(12.42) |  |
| Range | 15-102 | 17-99 | 15-102 |  | 13-99 | 15-95 | 13-99 |  |
| < 50 | 402.03 (11.88%) | 137.06 (11.41%) | 264.97 (12.15%) |  | 1616.07 (11.88%) | 470.34 (11.65%) | 1145.72 (11.98%) |  |
| 50 - 59 | 696.16 (20.58%) | 263.63 (21.94%) | 432.53 (19.83%) |  | 2831.78 (20.82%) | 846.96 (20.98%) | 1984.82 (20.75%) |  |
| 60 - 69 | 822.52 (24.31%) | 282.26 (23.49%) | 540.26 (24.76%) |  | 3337.44 (24.53%) | 989.26 (24.51%) | 2348.18 (24.54%) |  |
| ≥ 70 | 1462.45 (43.23%) | 518.57 (43.16%) | 943.88 (43.26%) |  | 5818.74 (42.77%) | 1730.35 (42.86%) | 4088.40 (42.73%) |  |
| **Sex** |  |  |  | -0.01 |  |  |  | 0.00 |
| Men | 1850.91 (54.71%) | 663.05 (55.18%) | 1187.86 (54.45%) |  | 7389.95 (54.32%) | 2199.12 (54.48%) | 5190.83 (54.26%) |  |
| Women | 1532.25 (45.29%) | 538.47 (44.82%) | 993.78 (45.55%) |  | 6214.08 (45.68%) | 1837.79 (45.52%) | 4376.29 (45.74%) |  |
| **Enrollee category** |  |  |  | 0.02 |  |  |  | 0.02 |
| EC1 | 257.69 (7.62%) | 92.71 (7.72%) | 164.98 (7.56%) |  | 1039.43 (7.64%) | 308.61 (7.64%) | 730.82 (7.64%) |  |
| EC2 | 943.48 (27.89%) | 343.52 (28.59%) | 599.96 (27.5%) |  | 3740.80 (27.5%) | 1116.90 (27.67%) | 2623.90 (27.43%) |  |
| EC3 | 1310.02 (38.72%) | 458.08 (38.12%) | 851.94 (39.05%) |  | 5367.42 (39.45%) | 1587.67 (39.33%) | 3779.75 (39.51%) |  |
| EC4 | 871.96 (25.77%) | 307.21 (25.57%) | 564.75 (25.89%) |  | 3456.38 (25.41%) | 1023.73 (25.36%) | 2432.65 (25.43%) |  |
| **Income** |  |  |  | 0.07 |  |  |  | 0.03 |
| Dependent | 1142.93 (33.78%) | 418.85 (34.86%) | 724.07 (33.19%) |  | 4654.36 (34.21%) | 1381.94 (34.23%) | 3272.42 (34.2%) |  |
| <15,000 | 662.25 (19.57%) | 228.46 (19.01%) | 433.79 (19.88%) |  | 2648.96 (19.47%) | 782.48 (19.38%) | 1866.47 (19.51%) |  |
| 15,000-24,999 | 1034.85 (30.59%) | 373.99 (31.13%) | 660.86 (30.29%) |  | 4099.95 (30.14%) | 1226.41 (30.38%) | 2873.54 (30.04%) |  |
| ≥ 25,000 | 543.13 (16.05%) | 180.22 (15%) | 362.91 (16.63%) |  | 2200.77 (16.18%) | 646.07 (16%) | 1554.69 (16.25%) |  |
| **Tumor location** |  |  |  | 0.08 |  |  |  | 0.02 |
| Left side | 1645.72 (48.64%) | 582.13 (48.45%) | 1063.59 (48.75%) |  | 6643.35 (48.83%) | 1944.57 (48.17%) | 4698.78 (49.11%) |  |
| Right side | 1317.82 (38.95%) | 469.01 (39.03%) | 848.81 (38.91%) |  | 5173.06 (38.03%) | 1567.75 (38.84%) | 3605.31 (37.68%) |  |
| Rectosigmoid | 381.84 (11.29%) | 137.38 (11.43%) | 244.46 (11.21%) |  | 1622.12 (11.92%) | 471.37 (11.68%) | 1150.75 (12.03%) |  |
| Unspecified | 37.78 (1.12%) | 13.00 (1.08%) | 24.78 (1.14%) |  | 165.50 (1.22%) | 53.21 (1.32%) | 112.29 (1.17%) |  |
| **Surgery type** |  |  |  | 0.1 |  |  |  | 0.03 |
| Open | 2767.75 (81.81%) | 1001.59 (83.36%) | 1766.17 (80.96%) |  | 11034.85 (81.11%) | 3310.09 (82%) | 7724.76 (80.74%) |  |
| Laprascopic | 499.12 (14.75%) | 158.90 (13.22%) | 340.23 (15.6%) |  | 2134.60 (15.69%) | 602.74 (14.93%) | 1531.86 (16.01%) |  |
| Unknown | 116.28 (3.44%) | 41.04 (3.42%) | 75.24 (3.45%) |  | 434.58 (3.19%) | 124.07 (3.07%) | 310.50 (3.25%) |  |
| **Tumor grade** |  |  |  | 0.05 |  |  |  | 0.00 |
| Well or moderately differentiated | 2900.22 (85.73%) | 1026.42 (85.43%) | 1873.80 (85.89%) |  | 11696.31 (85.98%) | 3482.98 (86.28%) | 8213.32 (85.85%) |  |
| Poorly differentiated | 365.65 (10.81%) | 132.50 (11.03%) | 233.15 (10.69%) |  | 1491.09 (10.96%) | 424.07 (10.5%) | 1067.02 (11.15%) |  |
| Unknown | 117.29 (3.47%) | 42.60 (3.55%) | 74.69 (3.42%) |  | 416.63 (3.06%) | 129.85 (3.22%) | 286.78 (3%) |  |
| **pT stage (AJCC 6th and 7th)** |  |  |  | 0.14 |  |  |  | 0.11 |
| 1 | 66.26 (1.96%) | 23.73 (1.98%) | 42.53 (1.95%) |  | 346.84 (2.55%) | 98.00 (2.43%) | 248.83 (2.6%) |  |
| 2 | 210.36 (6.22%) | 68.89 (5.73%) | 141.47 (6.48%) |  | 802.95 (5.9%) | 236.76 (5.86%) | 566.19 (5.92%) |  |
| 3 | 2369.96 (70.05%) | 848.90 (70.65%) | 1521.06 (69.72%) |  | 9550.69 (70.2%) | 2847.06 (70.53%) | 6703.63 (70.07%) |  |
| 4 | 658.75 (19.47%) | 235.80 (19.63%) | 422.95 (19.39%) |  | 2687.45 (19.75%) | 782.34 (19.38%) | 1905.11 (19.91%) |  |
| 0+Unknown | 77.83 (2.31%) | 24.20 (2.01%) | 53.63 (2.46%) |  | 216.11 (1.59%) | 72.75 (1.8%) | 143.36 (1.50%) |  |
| **pN stage (AJCC 6th and 7th)** |  |  |  | 0.06 |  |  |  | 0.08 |
| 1 | 2161.77 (63.9%) | 749.21 (62.36%) | 1412.55 (64.75%) |  | 8804.26 (64.72%) | 2616.04 (64.8%) | 6188.21 (64.68%) |  |
| 2 | 1144.73 (33.84%) | 428.75 (35.68%) | 715.98 (32.82%) |  | 4589.48 (33.74%) | 1351.61 (33.48%) | 3237.87 (33.84%) |  |
| 0+Unknown | 76.67 (2.27%) | 23.56 (1.87%) | 53.1 (2.43%) |  | 210.29 (1.54%) | 69.25 (1.72%) | 141.04 (1.47%) |  |
| **Surgical margins** |  |  |  | 0.06 |  |  |  | 0.08 |
| No | 3141.00 (92.84%) | 1110.22 (92.4%) | 2030.79 (93.09%) |  | 12793.59 (94.04%) | 3797.38 (94.07%) | 8996.21 (94.03%) |  |
| Yes | 89.18 (2.64%) | 36.48 (3.04%) | 52.70 (2.42%) |  | 351.37 (2.58%) | 96.69 (2.4%) | 254.68 (2.66%) |  |
| Unknown | 152.97 (4.52%) | 54.82 (4.56%) | 98.15 (4.5%) |  | 459.06 (3.37%) | 142.84 (3.54%) | 316.23 (3.31%) |  |
| **Charlson comorbidity score** |  |  |  | 0.07 |  |  |  | 0.02 |
| 0 | 757.99 (22.4%) | 293.18 (24.4%) | 464.82 (21.31%) |  | 3168.03 (23.29%) | 933.09 (23.11%) | 2234.94 (23.36%) |  |
| 1 | 760.65 (22.48%) | 259.07 (21.56%) | 501.58 (22.99%) |  | 3208.77 (23.59%) | 934.05 (23.14%) | 2274.72 (23.78%) |  |
| ≥ 2 | 1864.52 (55.11%) | 649.28 (54.04%) | 1215.24 (55.7%) |  | 7227.22 (53.13%) | 2169.77 (53.75%) | 5057.45 (52.86%) |  |
| **Comorbidity** |  |  |  |  |  |  |  |  |
| Prior cancer history | 346.58 (10.24%) | 128.07 (10.66%) | 218.51 (10.02%) | -0.02 | 1361.07 (10%) | 410.44 (10.17%) | 950.63 (9.94%) | -0.01 |
| Ischemic heart disease | 1319.50 (39%) | 480.53 (39.99%) | 838.97 (38.46%) | -0.03 | 5135.91 (37.75%) | 1530.06 (37.9%) | 3605.86 (37.69%) | 0.00 |
| Stroke | 605.17 (17.89%) | 224.76 (18.71%) | 380.41 (17.44%) | -0.03 | 2348.78 (17.27%) | 682.80 (16.91%) | 1665.99 (17.41%) | 0.01 |
| Diabetes mellitus | 1235.77 (36.53%) | 450.51 (37.5%) | 785.26 (35.99%) | -0.03 | 5126.18 (37.68%) | 1542.81 (38.22%) | 3583.36 (37.45%) | -0.02 |
| Hypertension | 2092.31 (61.84%) | 766.58 (63.8%) | 1325.73 (60.77%) | -0.06 | 8316.38 (61.13%) | 2477.69 (61.38%) | 5838.69 (61.03%) | -0.01 |
| Dyspilidemia | 1580.88 (46.73%) | 554.62 (46.16%) | 1026.26 (47.04%) | 0.02 | 6370.57 (46.83%) | 1850.15 (45.83%) | 4520.42 (47.25%) | 0.03 |
| Chronic kidney disease | 1078.77 (31.89%) | 362.44 (30.16%) | 716.34 (32.83%) | 0.06 | 4277.64 (31.44%) | 1267.41 (31.4%) | 3010.23 (31.46%) | 0.00 |
| Chronic liver disease | 1382.09 (40.85%) | 474.59 (39.5%) | 907.50 (41.6%) | 0.04 | 5468.91 (40.2%) | 1615.42 (40.02%) | 3853.49 (40.28%) | 0.01 |
| **Medications** |  |  |  |  |  |  |  |  |
| Aspirin | 1039.69 (30.73%) | 357.04 (29.72%) | 682.65 (31.29%) | 0.03 | 4156.22 (30.55%) | 1231.59 (30.51%) | 2924.64 (30.57%) | 0.00 |
| Metformin | 634.43 (18.75%) | 221.06 (18.4%) | 413.37 (18.95%) | 0.01 | 2593.91 (19.07%) | 769.33 (19.06%) | 1824.59 (19.07%) | 0.00 |
| Statin | 734.49 (21.71%) | 262.17 (21.82%) | 472.32 (21.65%) | 0 | 2923.55 (21.49%) | 853.61 (21.15%) | 2069.94 (21.64%) | 0.01 |

SIPTW: stabilized inverse probability of treatment weighting

SMD: standardized mean differences

**Supplementary file 4.** (**A) Number of patients with stage III colon cancer receiving different types of adjuvant treatment by calendar year. (B) The percentage distribution of patients with stage III colon cancer receiving different types of adjuvant treatment by calendar year.**

**
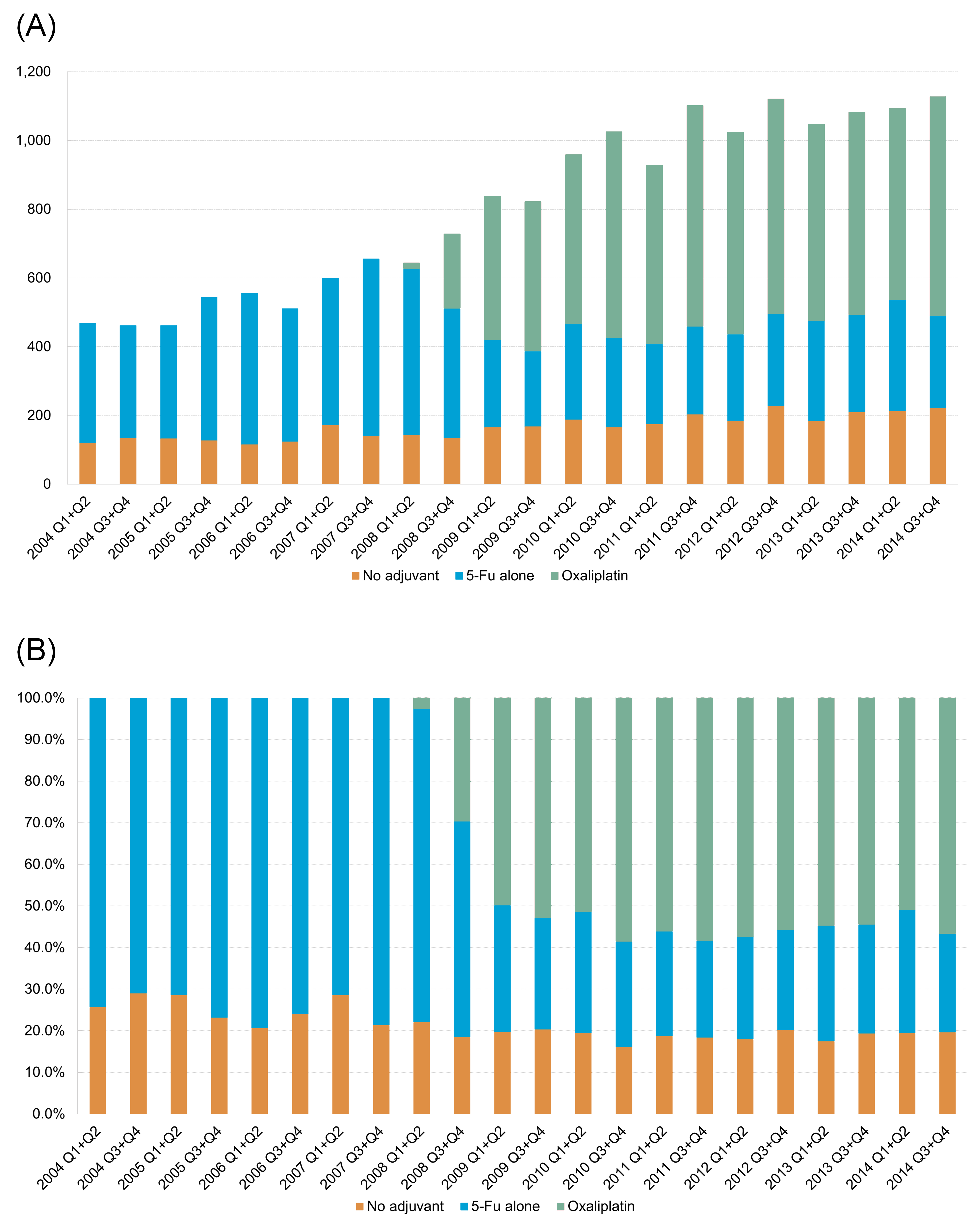
**

**Supplementary file 5. Survival outcomes by calendar year before and after oxaliplatin reimbursement for subgroups. (A) Three-year DFS rates for age < 70 years old, (B) Three-year DFS rates for age > 70 years old, (C) Five-year OS rates for age < 70 years old, (D) Five-year OS rates for age > 70 years old, (E) Three-year DFS rates for T1-T3 and N1, (F) Three-year DFS rates for T4 or N2, (G) Five-year OS rates for T1-T3 and N1, (H) Five-year OS rates for T4 or N2, (I) Three-year DFS rates for oxaliplatin < 6 cycles, (J) Three-year DFS rates for oxaliplatin > 6 cycles, (K) Five-year OS rates for oxaliplatin < 6 cycles, (L) Five-year OS rates for oxaliplatin > 6 cycles.**

**
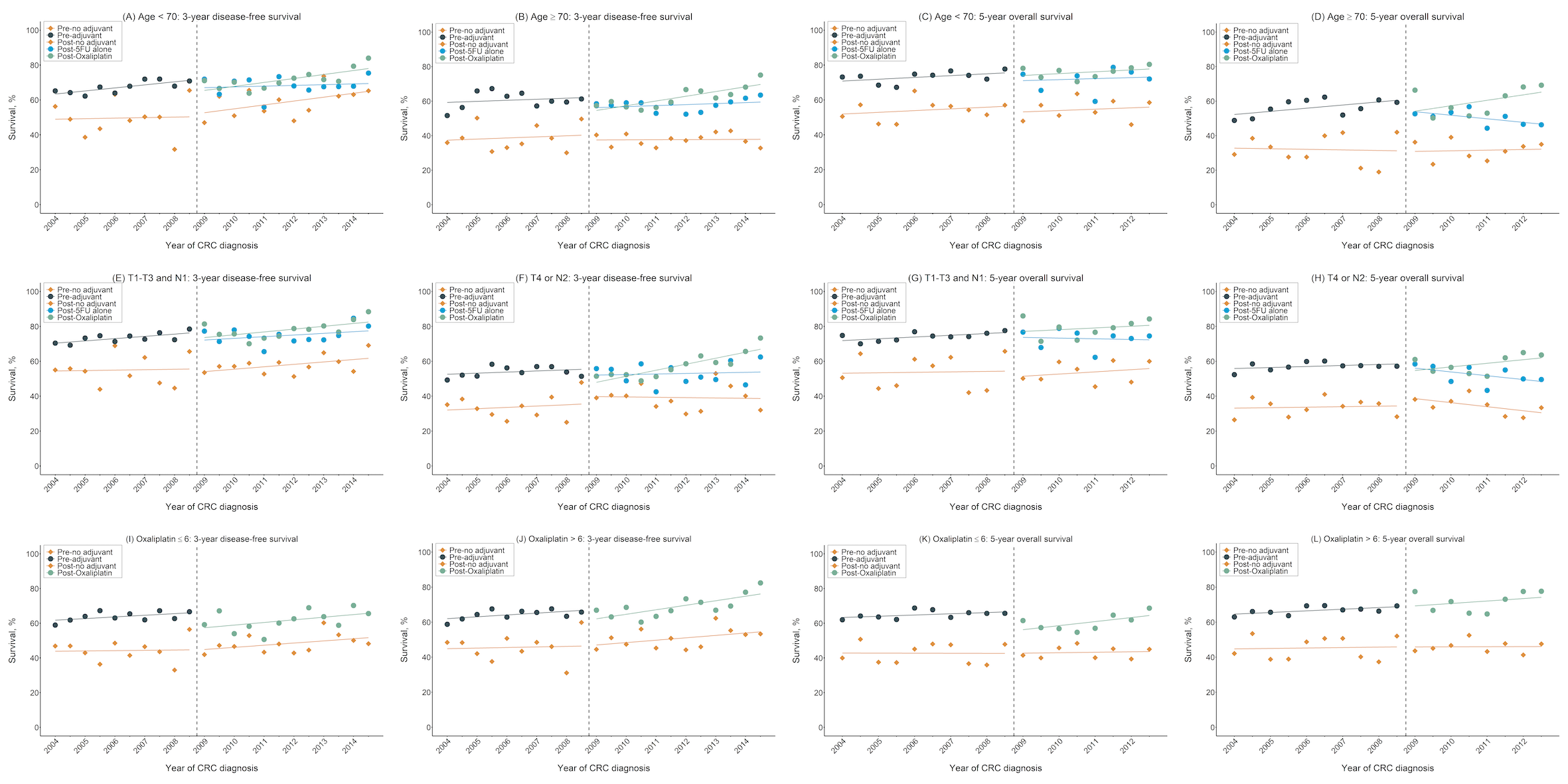
**

**Supplement file 6. The CITS result: Subgroup analysis by age**

|  | **Age<70** | | | |  | **Age>=70** | | | |
| --- | --- | --- | --- | --- | --- | --- | --- | --- | --- |
|  | **3-year disease-free survival** | | **5-year overall survival** | |  | **3-year disease-free survival** | | **5-year overall survival** | |
|  | Coefficient (95% CI) | p value | Coefficient (95% CI) | p value |  | Coefficient (95% CI) | p value | Coefficient (95% CI) | p value |
| **Overall** |  |  |  |  |  |  |  |  |  |
| **Pre-intervention: adjuvant group vs. no adjuvant group** |  |  |  |  |  |  |  |  |  |
| Level difference (β_4_) | 0.187(0.089,0.285) | 0.0005 | 0.242(0.156,0.328) | <.0001 |  | 0.224(0.159,0.29) | <.0001 | 0.237(0.158,0.316) | <.0001 |
| Slope difference (β_5_) | 0.004(-0.014,0.022) | 0.661 | 0.001(-0.015,0.017) | 0.9064 |  | 0.005(-0.007,0.018) | 0.3783 | 0.01(-0.005,0.024) | 0.1948 |
| **Post-intervention: adjuvant group vs. no adjuvant group** |  |  |  |  |  |  |  |  |  |
| Level difference in difference (β_6_) | -0.078(-0.223,0.067) | 0.2848 | -0.056(-0.194,0.082) | 0.4115 |  | -0.054(-0.151,0.044) | 0.2708 | -0.065(-0.191,0.061) | 0.3001 |
| Slope difference in difference (β_7_) | -0.007(-0.03,0.016) | 0.5166 | 0.003(-0.025,0.03) | 0.854 |  | 0.001(-0.015,0.016) | 0.9287 | -0.009(-0.035,0.016) | 0.4685 |
| **Oxaliplatin** |  |  |  |  |  |  |  |  |  |
| **Pre-intervention: adjuvant group vs. no adjuvant group** |  |  |  |  |  |  |  |  |  |
| Level difference (β_4_) | 0.187(0.089,0.284) | 0.0004 | 0.242(0.157,0.327) | <.0001 |  | 0.224(0.157,0.292) | <.0001 | 0.237(0.147,0.326) | <.0001 |
| Slope difference (β_5_) | 0.004(-0.014,0.022) | 0.6603 | 0.001(-0.015,0.017) | 0.9057 |  | 0.005(-0.007,0.018) | 0.3924 | 0.01(-0.007,0.026) | 0.2523 |
| **Post-intervention: adjuvant group vs. no adjuvant group** |  |  |  |  |  |  |  |  |  |
| Level difference in difference (β_6_) | -0.086(-0.231,0.059) | 0.2381 | -0.058(-0.195,0.078) | 0.3902 |  | -0.078(-0.178,0.022) | 0.124 | -0.068(-0.211,0.076) | 0.3427 |
| Slope difference in difference (β_7_) | -0.006(-0.029,0.017) | 0.6089 | 0.004(-0.023,0.032) | 0.7519 |  | 0.009(-0.007,0.025) | 0.2482 | 0.005(-0.024,0.034) | 0.733 |

CITS: controlled interrupted time series; *β_4_*: the level difference of the outcome at the beginning of the pre-intervention period between the control and the treated group; *β_5:_* the slope difference of the outcome in the pre-intervention period between the control and the treated group; *β_6:_* the level change of the outcome immediately after intervention started between the control and the treated group (ie. difference-in-difference of the level); *β_7:_* the slope change of the outcome after post-intervention period between the control and the treated group (ie. difference-in-difference of the slope); Please see eMethods in the supplement for the explanation of β_i_

**Supplement file 7. The CITS result: Subgroup analysis by stage**

|  | **T1-T3 and N1** | | | |  | **T4 or N2** | | | |
| --- | --- | --- | --- | --- | --- | --- | --- | --- | --- |
|  | **3-year disease-free survival** | | **5-year overall survival** | |  | **3-year disease-free survival** | | **5-year overall survival** | |
|  | Coefficient (95% CI) | p value | Coefficient (95% CI) | p value |  | Coefficient (95% CI) | p value | Coefficient (95% CI) | p value |
| **Overall** |  |  |  |  |  |  |  |  |  |
| **Pre-intervention: adjuvant group vs. no adjuvant group** |  |  |  |  |  |  |  |  |  |
| Level difference (β_4_) | 0.212(0.13,0.294) | <.0001 | 0.297(0.205,0.388) | <.0001 |  | 0.248(0.185,0.311) | <.0001 | 0.303(0.251,0.355) | <.0001 |
| Slope difference (β_5_) | 0.007(-0.009,0.022) | 0.3825 | 0.003(-0.014,0.02) | 0.7298 |  | 0.006(-0.006,0.018) | 0.3054 | 0.004(-0.005,0.014) | 0.374 |
| **Post-intervention: adjuvant group vs. no adjuvant group** |  |  |  |  |  |  |  |  |  |
| Level difference in difference (β_6_) | 0.019(-0.102,0.14) | 0.7544 | 0.037(-0.11,0.184) | 0.6137 |  | -0.135(-0.229,-0.041) | 0.0062 | -0.105(-0.188,-0.022) | 0.0154 |
| Slope difference in difference (β_7_) | -0.008(-0.027,0.011) | 0.4098 | -0.005(-0.034,0.025) | 0.7562 |  | 0.01(-0.005,0.025) | 0.1842 | 0.014(-0.002,0.031) | 0.0883 |
| **Oxaliplatin** |  |  |  |  |  |  |  |  |  |
| **Pre-intervention: adjuvant group vs. no adjuvant group** |  |  |  |  |  |  |  |  |  |
| Level difference (β_4_) | 0.212(0.131,0.293) | <.0001 | 0.297(0.205,0.388) | <.0001 |  | 0.248(0.182,0.313) | <.0001 | 0.303(0.247,0.359) | <.0001 |
| Slope difference (β_5_) | 0.007(-0.009,0.022) | 0.3812 | 0.003(-0.014,0.02) | 0.729 |  | 0.006(-0.006,0.018) | 0.3209 | 0.004(-0.006,0.015) | 0.4059 |
| **Post-intervention: adjuvant group vs. no adjuvant group** |  |  |  |  |  |  |  |  |  |
| Level difference in difference (β_6_) | 0.039(-0.082,0.16) | 0.5186 | 0.086(-0.06,0.233) | 0.2391 |  | -0.132(-0.229,-0.035) | 0.0091 | -0.081(-0.17,0.008) | 0.074 |
| Slope difference in difference (β_7_) | -0.006(-0.025,0.013) | 0.5067 | -0.004(-0.033,0.026) | 0.7947 |  | 0.015(-0.001,0.03) | 0.0594 | 0.02(0.002,0.038) | 0.0297 |

CITS: controlled interrupted time series; *β_4_*: the level difference of the outcome at the beginning of the pre-intervention period between the control and the treated group; *β_5:_* the slope difference of the outcome in the pre-intervention period between the control and the treated group; *β_6:_* the level change of the outcome immediately after intervention started between the control and the treated group (ie. difference-in-difference of the level); *β_7:_* the slope change of the outcome after post-intervention period between the control and the treated group (ie. difference-in-difference of the slope); Please see eMethods in the supplement for the explanation of β_i_

**Supplement file 8. The CITS result: Subgroup analysis by cycle of oxaliplatin use**

|  | **Oxaliplatin<6** | | | |  | **Oxaliplatin>=6** | | | |
| --- | --- | --- | --- | --- | --- | --- | --- | --- | --- |
|  | **3-year DFS** | | **5-year OS** | |  | **3-year DFS** | | **5-year OS** | |
|  | Coefficient (95% CI) | p value | Coefficient (95% CI) | p value |  | Coefficient (95% CI) | p value | Coefficient (95% CI) | p value |
| **Oxaliplatin** |  |  |  |  |  |  |  |  |  |
| **Pre-intervention: adjuvant group vs. no adjuvant group** |  |  |  |  |  |  |  |  |  |
| Level difference (β_4_) | 0.215(0.148,0.282) | <.0001 | 0.281(0.218,0.345) | <.0001 |  | 0.215(0.154,0.276) | <.0001 | 0.28(0.214,0.346) | <.0001 |
| Slope difference (β_5_) | 0.007(-0.006,0.02) | 0.2681 | 0.005(-0.007,0.017) | 0.4048 |  | 0.007(-0.005,0.018) | 0.2314 | 0.005(-0.007,0.018) | 0.3837 |
| **Post-intervention: adjuvant group vs. no adjuvant group** |  |  |  |  |  |  |  |  |  |
| Level difference in difference (β_6_) | -0.098(-0.197,0.002) | 0.054 | -0.096(-0.197,0.005) | 0.0624 |  | -0.054(-0.144,0.037) | 0.2378 | 0.006(-0.099,0.111) | 0.9112 |
| Slope difference in difference (β_7_) | -0.001(-0.016,0.015) | 0.94 | 0.006(-0.014,0.027) | 0.5202 |  | 0.001(-0.013,0.015) | 0.8713 | 0.001(-0.02,0.022) | 0.9079 |

CITS: controlled interrupted time series; *β_4_*: the level difference of the outcome at the beginning of the pre-intervention period between the control and the treated group; *β_5:_* the slope difference of the outcome in the pre-intervention period between the control and the treated group; *β_6:_* the level change of the outcome immediately after intervention started between the control and the treated group (ie. difference-in-difference of the level); *β_7:_* the slope change of the outcome after post-intervention period between the control and the treated group (ie. difference-in-difference of the slope); Please see eMethods in the supplement for the explanation of β_i_

**Supplement file 9.** **Survival outcomes by calendar year before and after oxaliplatin reimbursement, restricting to the following (sensitivity analysis). (A) Three-year DFS rates for those with no prior cancer history, (B) Five-year OS rates for those with no prior cancer history, (C) Three-year DFS rates excluding the one-year transition period, (D) Five-year OS rates excluding the one-year transition period, (E) Three-year DFS rates excluding patients receiving biweekly fluoropyrimidine treatment before the intervention, (F) Five-year OS rates excluding patients receiving biweekly fluoropyrimidine treatment before the intervention.**

**
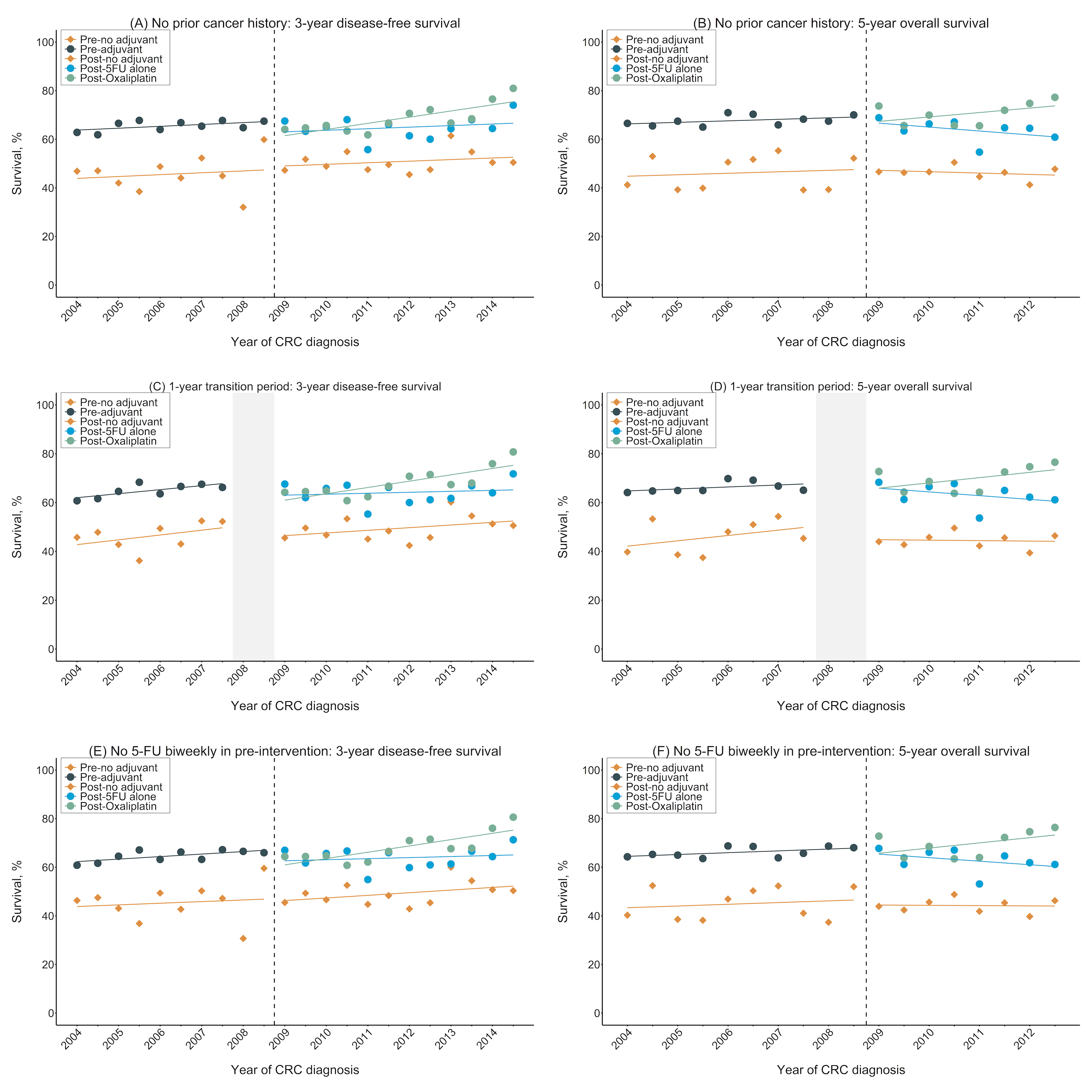
**

**Supplement file 10. The CITS result: Sensitivity analysis**

|  | No prior cancer history: DFS | | No prior cancer history: OS | |  | 1-year transition period: DFS | | 1-year transition period: OS | |  | No 5-Fu biweekly in pre-intervention: DFS | | No 5-Fu biweekly in pre-intervention: OS | |
| --- | --- | --- | --- | --- | --- | --- | --- | --- | --- | --- | --- | --- | --- | --- |
|  | Coefficient (95% CI) | p value | Coefficient (95% CI) | p value |  | Coefficient (95% CI) | p value | Coefficient (95% CI) | p value |  | Coefficient (95% CI) | p value | Coefficient (95% CI) | p value |
| **Overall** |  |  |  |  |  |  |  |  |  |  |  |  |  |  |
| **Pre-intervention: adjuvant group vs. no adjuvant group** |  |  |  |  |  |  |  |  |  |  |  |  |  |  |
| Level difference (β_4_) | 0.199(0.118,0.28) | <.0001 | 0.215(0.138,0.293) | <.0001 |  | 0.202(0.137,0.266) | <.0001 | 0.219(0.147,0.291) | <.0001 |  | 0.185(0.101,0.268) | <.0001 | 0.211(0.136,0.287) | <.0001 |
| Slope difference (β_5_) | 0(-0.015,0.015) | 0.9998 | 0(-0.014,0.015) | 0.99 |  | -0.005(-0.018,0.007) | 0.3711 | -0.004(-0.017,0.009) | 0.5501 |  | 0.002(-0.014,0.017) | 0.817 | 0(-0.014,0.014) | 0.9756 |
| **Post-intervention: adjuvant group vs. no adjuvant group** |  |  |  |  |  |  |  |  |  |  |  |  |  |  |
| Level difference in difference (β_6_) | -0.068(-0.188,0.053) | 0.2618 | -0.02(-0.144,0.104) | 0.7437 |  | 0.006(-0.09,0.102) | 0.9 | 0.03(-0.085,0.145) | 0.6007 |  | -0.049(-0.173,0.075) | 0.4296 | -0.004(-0.125,0.117) | 0.9476 |
| Slope difference in difference (β_7_) | 0.006(-0.013,0.025) | 0.5101 | 0.008(-0.017,0.033) | 0.5374 |  | 0.009(-0.006,0.024) | 0.2278 | 0.011(-0.012,0.034) | 0.352 |  | 0.002(-0.018,0.022) | 0.8292 | 0.006(-0.018,0.031) | 0.6022 |
| **Oxaliplatin** |  |  |  |  |  |  |  |  |  |  |  |  |  |  |
| **Pre-intervention: adjuvant group vs. no adjuvant group** |  |  |  |  |  |  |  |  |  |  |  |  |  |  |
| Level difference (β_4_) | 0.199(0.117,0.281) | <.0001 | 0.215(0.136,0.295) | <.0001 |  | 0.202(0.135,0.268) | <.0001 | 0.219(0.145,0.293) | <.0001 |  | 0.185(0.1,0.269) | <.0001 | 0.211(0.133,0.29) | <.0001 |
| Slope difference (β_5_) | 0(-0.015,0.015) | 0.9998 | 0(-0.015,0.015) | 0.9902 |  | -0.005(-0.018,0.007) | 0.3817 | -0.004(-0.018,0.01) | 0.5626 |  | 0.002(-0.014,0.018) | 0.8197 | 0(-0.014,0.015) | 0.9763 |
| **Post-intervention: adjuvant group vs. no adjuvant group** |  |  |  |  |  |  |  |  |  |  |  |  |  |  |
| Level difference in difference (β_6_) | -0.074(-0.196,0.048) | 0.2249 | -0.015(-0.142,0.112) | 0.8106 |  | -0.002(-0.1,0.096) | 0.9659 | 0.032(-0.087,0.151) | 0.5873 |  | -0.056(-0.181,0.07) | 0.3765 | -0.001(-0.125,0.124) | 0.9924 |
| Slope difference in difference (β_7_) | 0.009(-0.01,0.029) | 0.329 | 0.012(-0.014,0.037) | 0.3513 |  | 0.013(-0.003,0.029) | 0.1002 | 0.016(-0.008,0.04) | 0.1902 |  | 0.006(-0.014,0.026) | 0.5613 | 0.011(-0.014,0.036) | 0.3753 |

CITS: controlled interrupted time series; *β_4_*: the level difference of the outcome at the beginning of the pre-intervention period between the control and the treated group; *β_5:_* the slope difference of the outcome in the pre-intervention period between the control and the treated group; *β_6:_* the level change of the outcome immediately after intervention started between the control and the treated group (ie. difference-in-difference of the level); *β_7:_* the slope change of the outcome after post-intervention period between the control and the treated group (ie. difference-in-difference of the slope); Please see eMethods in the supplement for the explanation of β_i_

**Supplement file 11. Joinpoint analysis of 3-year disease-free survival (DFS) and 5-year overall survival (OS) for patients with stage III colon cancer**

|  |  | No adjuvant | | | Adjuvant | | |
| --- | --- | --- | --- | --- | --- | --- | --- |
|  |  | SAPC | SE | p value | SAPC | SE | p value |
| 3-year DFS | 2004-2013 | 0.003872 | 0.002074 | 0.076615 | 0.001561 | 0.000975 | 0.12778 |
|  | 2014 |  |  |  | 0.055366 | 0.032911 | 0.110789 |
| 5-year OS | 2004-2014 | 0.000068 | 0.002333 | 0.9772 | 0.002156 | 0.001322 | 0.122473 |

SAPC: semi-annual percent change

SE: standard error
